# Pregnancy success of lactating Holstein cows after a single administration of a sustained-release formulation of recombinant bovine somatotropin

**DOI:** 10.1186/1746-6148-4-22

**Published:** 2008-06-26

**Authors:** A Bell, OA Rodríguez, LA de Castro e Paula, MB Padua, J Hernández-Cerón, CG Gutiérrez, A De Vries, PJ Hansen

**Affiliations:** 1 Department of Animal Sciences, University of Florida, Gainesville, Florida 32611-0910, USA; 2 Departamento de Reproducción, Facultad de Medicina Veterinaria y Zootecnia, Universidad Nacional Autónoma de México, Ciudad Universitaria, DF, CP 04510, México

## Abstract

**Background:**

Results regarding the use of bovine somatotropin for enhancing fertility in dairy cattle are variable. Here, the hypothesis was tested that a single injection of a sustained-release preparation of bovine somatotropin (bST) during the preovulatory period would improve pregnancy success of lactating dairy cows at first service.

**Results:**

The first experiment was conducted in a temperate region of Mexico. Cows inseminated following natural estrus or timed artificial insemination were given a single injection of bST or a placebo injection at insemination (n = 100 cows per group). There was no significant difference between bST and control groups in the proportion of inseminated cows diagnosed pregnant (29 vs 31% pregnant). The second experiment was performed during heat stress in Florida. Cows were subjected to an ovulation synchronization regimen for first insemination. Cows treated with bST received a single injection at 3 days before insemination. Controls received no additional treatment. As expected, bST did not increase vaginal temperature. Treatment with bST did not significantly increase the proportion of inseminated cows diagnosed pregnant although it was numerically greater for the bST group (24.2% vs 17.8%, 124–132 cows per group). There was a tendency (*p *= 0.10) for a smaller percent of control cows to have high plasma progesterone concentrations (≥ 1 ng/ml) at Day 7 after insemination than for bST-treated cows (72.6 vs 81.1%). When only cows that were successfully synchronized were considered, the magnitude of the absolute difference in the percentage of inseminated cows that were diagnosed pregnant between bST and control cows was reduced (24.8 vs 22.4% pregnant for bST and control).

**Conclusion:**

Results failed to indicate a beneficial effect of bST treatment on fertility of lactating dairy cows.

## Background

Treatment of lactating cows with recombinant bovine somatotropin (bST) can increase milk yield in thermoneutral and heat-stress environments [1-6]. The consequences of bST treatment for fertility are not clear. In early studies, cows treated with bST had an increased calving to conception interval that was attributed, at least in part, to increased anestrus [7,8]. In contrast, bST either increased [9,10] or had no effect [6,11,12] on pregnancy rate per insemination when cows were inseminated using a timed artificial insemination (TAI) protocol. Treatment with bST increased pregnancy rate per insemination for repeat-breeder cows bred at estrus [13].

Bovine ST might be particularly effective at increasing fertility during periods of heat stress. This is because insulin-like growth factor-1 (IGF-1), whose secretion is stimulated by bST [6,14], has thermoprotective properties and can reduce the effect of elevated temperature on development and apoptosis of cultured bovine embryos [15,16]. Indeed, the proportion of heat-stressed lactating cows receiving an in vitro produced embryo that became pregnant was higher when embryos were cultured in IGF-1 before transfer [17,18]. This effect of IGF-1 was not seen for cows in cool weather [18].

Despite the promise of using bST in the summer to increase IGF-1 and improve embryonic resistance to heat stress, there was no effect of bST on pregnancy rate in the one experiment that examined bST effects on fertility during heat stress [6]. In this experiment, cows received bST every 14 days beginning 13 days before TAI. The failure to observe an increase in fertility caused by bST may have been because bST also increased body temperature.

The hypothesis of the current study was that a single injection of a sustained-release preparation of bST during the preovulatory period could improve fertility of lactating cows. The rationale for the hypothesis was that bST, directly or through an increase in IGF-1 secretion, would increase fertility by exerting beneficial effects on follicular function [19,20], oocyte function [21,22] and embryonic development [23,24]. In heat–stressed cows, bST would also be thermoprotective towards the embryo through actions of IGF-1 [15,16]. Treatment with bST was limited to a single injection around the time of ovulation to exert effects on late follicular development, ovulation, and early embryonic development while avoiding milk yield responses during the peri-ovulatory period that could enhance hyperthermia or otherwise limit the possible beneficial effect of bST on fertility.

## Results

### Effects of bST at insemination in cows in a temperate climate (Experiment 1)

As shown in Table 1, there was no effect of bST on the proportion of cows that were pregnant following insemination. In addition, there was no significant effect of breeding at estrus vs TAI (Table 1), parity or days in milk (results not shown) on pregnancy rate per insemination.

**Table 1:** Effect of bovine somatotropin administered at insemination on the proportion of cows inseminated at first service that became pregnant (Experiment 1).^a^

	Treatment^b^				
				
	Control	bST	*p*	AOR	CI
Cows bred at estrus	20/65 (31%)	19/65 (29%)			
Cows subject to TAI	11/35 (31%)	10/35 (29%)			
Total	31/100 (31%)	29/100 (29%)	NS	0.96	0.69–1.32

^a ^Abbreviations are bST; bovine somatotropin; AOR, adjusted odds ratio; CI, 95% confidence interval.

^b ^Data are the number of cows pregnant/total cows inseminated and, in parentheses, percent pregnant.

### Effects of bST in cows exposed to heat stress (Experiment 2)

**Figure 1:**
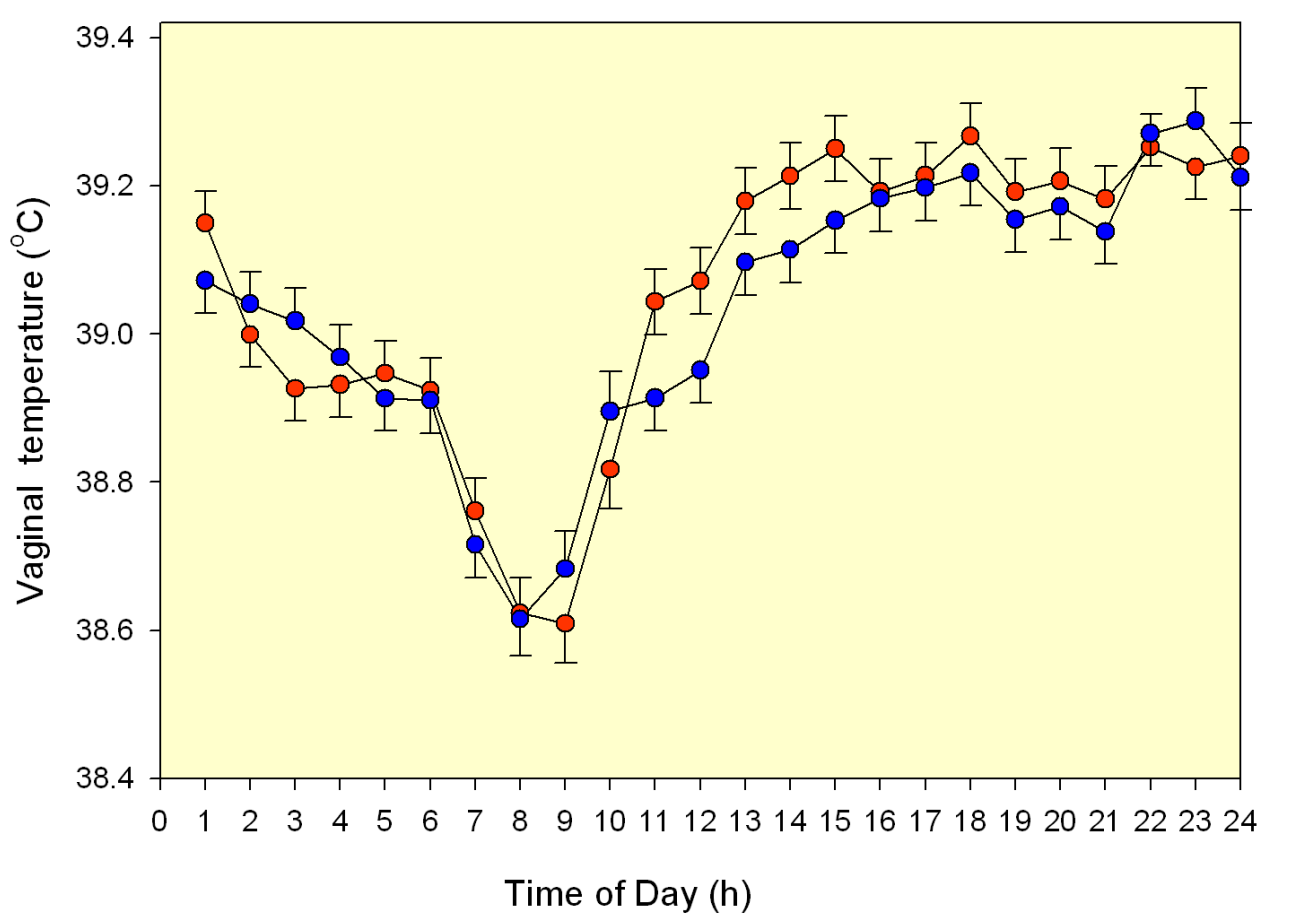
**Effect of bovine somatotropin on vaginal temperature (Experiment 2).** Data represent least-squares means ± SEM. There was an effect of time (*p *< 0.001) but no difference between control (blue circles) and bST-treated cows (red circles) and no time × treatment interaction.

The 24-h pattern of vaginal temperatures is shown in Fig. 1. Time of day affected vaginal temperature (*p *< 0.001). The peak temperature (least-squares mean) for both groups was 39.3°C and occurred at 23:00 h for control cows and 18:00 h for bST-treated cows. The nadir, 38.6°C for both control and bST-treated cows, occurred at 08:00 and 09:00 h, respectively. Vaginal temperature was not affected by bST treatment, day relative to TAI, or interactions between these main effects and other variables.

Body condition score at TAI was not different between control and bST-treated cows (2.52 ± 0.03 vs 2.54 ± 0.03).

Cows were considered to be successfully synchronized if progesterone concentration on the day of TAI (Day 0) was < 1 ng/ml and progesterone concentration on Day 7 after TAI was ≥ 1 ng/ml. Using this criterion, there was a tendency (*p *= 0.100) for a lower percent of control cows to be synchronized than bST-treated cows (77.3 vs 86.1%; Table 2). Further examination of progesterone concentrations revealed that, regardless of treatment, over 90% of cows had low progesterone concentrations (< 1 ng/ml) at Day 0. However, there was a tendency (*p *= 0.101) for a smaller percent of control cows to have high progesterone (≥ 1 ng/ml) at Day 7 than for bST-treated cows (72.6 vs 81.1%; Table 2). Thus, a smaller percent of cows ovulated in response to TAI for control cows than for bST-treated cows.

**Table 2:** Effect of bovine somatotropin administered 3 days before insemination on plasma progesterone concentrations on Day 0 and 7 relative to timed artificial insemination and on the percent of cows successfully synchronized (Experiment 2).^a^

	Treatment^b^				
				
	Control	bST	*p*	AOR	CI
Low progesterone (< 1 ng/ml) on Day 0	112/124 (90.3%)	123/132 (93.2%)	NS	1.45	0.58 to 3.58
High progesterone (≥ 1 ng/ml) on Day 7	90/124 (72.6%)	107/132 (81.1%)	0.10	1.66	0.91 to 3.03
Synchronized (low on d 0 and high on Day 7)	85/110 (77.3%)	105/122 (86.1%)	0.10	1.78	0.89 to 3.58

^a ^Abbreviations are bST; bovine somatotropin; AOR, adjusted odds ratio; CI, 95% confidence interval; NS, non-significant

^b ^Data are the number of cows with that classification/total cows inseminated and, in parentheses, percent.

Body condition score (BCS) at TAI also affected the successful synchronization rate (*p *= 0.03). Cows with BCS ≥ 2.5 were more likely to have been successfully synchronized (125/152, 87.5%) than cows with BCS < 2.5 (57/80, 71.3%) [adjusted odds ratio (AOR) = 0.36, 95% confidence interval (CI) = 0.18 ± 0.71].

Results for pregnancy following TAI are presented in Table 3. Among all cows, the percent of cows pregnant following TAI was not statistically affected by treatment. Numerically, the percent of inseminated cows that was pregnant was lower for control cows (17.8%) than for cows treated with bST (24.2%). This nonsignificant difference between groups was diminished in magnitude when the percent of inseminated cows that were pregnant was calculated for the subset of cows that were successfully synchronized (22.4 vs 24.8%, treatment effect, *p *> 0.10).

**Table 3:** Effect of bovine somatotropin administered 3 days before insemination on the proportion of cows pregnant after first service (Experiment 2).^a^

	Treatment^b^				
				
	Control	bST	*p*	AOR	CI
All cows	22/124 (17.8%)	32/132 (24.2%)	NS	1.50	0.81 to 2.76
Successfully synchronized cows ^c^	19/85 (22.4%)	26/105 (24.8%)	NS	1.12	0.51 to 2.20

^a ^Abbreviations are bST; bovine somatotropin; AOR, adjusted odds ratio; CI, confidence interval; NS, non-significant

^b ^Data are the number of cows pregnant/total cows inseminated and, in parentheses, percent pregnant.

^c^Cows where progesterone concentration on the day of timed artificial insemination (Day 0) was < 1 ng/ml and progesterone concentration on Day 7 after TAI was ≥ 1 ng/ml.

## Discussion

Results from the present study failed to support the hypothesis that administration of a sustained-release form of bST near the time of insemination improves fertility of lactating dairy cows. The hypothesis that bST improves fertility was tested independently at two different locations, with two different commercial bST preparations and using different breeding procedures. In the first experiment, conducted in the absence of heat stress and with most cows bred at natural estrus, there was no significant effect of bST on the percent of cows pregnant following first service. The preparation of bST used in the first experiment also contained vitamin E. Administration of vitamin E at insemination was without effect on pregnancy rate in lactating cows [25]. In the second experiment, where cows were heat-stressed and where TAI was the sole method for breeding cows, there was a numerical increase in the percent of cows pregnant following insemination for cows treated with bST as compared to control cows. However, this difference was not statistically different. Moreover, the numerical increase associated with bST was associated with effects on the response to ovulation synchronization rather than to effects on fertilization rate or embryonic survival. Specifically, bST tended to improve the percent of cows that ovulated after the ovulation synchronization protocol. The difference in percent of cows pregnant after TAI between control and bST-treated cows was reduced when only successfully-synchronized cows were considered.

Experiments to evaluate the beneficial effects of bST on ability of cattle to establish and maintain pregnancy after insemination in cattle have yielded variable results. Administration of a single injection of bST at insemination did not improve pregnancy rate per insemination in cows (largely beef cows) or beef heifers [26]. However, a single injection of bST at estrus increased the percent of repeat-breeder dairy cows pregnant following insemination [13]. Injections of bST increased pregnancy success in dairy cows bred to TAI in some studies [9,10] but not in others [6,11,12]. Differences in treatment regimens, product formulation, reproductive management, cow type and issues related to sample size could explain some of this variation. For example, bST improved pregnancy success for cows subjected to a Presynch-Ovsynch synchronization protocol but did not cause improvement for cows receiving an Ovsynch protocol without presynchronization [10]. Taken together, the lack of a consistent fertility-promoting effect of bST would contraindicate broad use of bST to improve fertility in lactating cows. Additional work is needed to confirm its efficacy in selected populations of cows, for example in repeat-breeder cows [13].

It was hypothesized that cows exposed to heat stress would be particularly likely to benefit from administration of bST because IGF-1, which is released in response to bST, can protect bovine preimplantation embryos from the deleterious effects of elevated temperature on development [13,14]. Nonetheless, bST did not improve fertility in Experiment 2 despite the fact that cows experienced vaginal temperatures characteristic of hyperthermia (greater than 38.6°C) throughout most of the day. There are several explanations for the lack of effect of bST during heat stress. For instance, it is possible that bST was not administered until damage to the oocyte had already occurred. Heat stress can compromise the follicle somewhere between 20–50 days before ovulation [27] and administration of bST three days before ovulation, as performed here, would probably not reverse oocyte damage occurring earlier in follicular development. The bST was not administered earlier relative to ovulation in Experiment 2 because of the desire to avoid the effect of bST on milk yield that could possibly result in increased body temperature [5]. In fact, the bST treatment did not increase vaginal temperature. Another possibility is that early embryos are refractory to the thermoprotective actions of IGF-1. The only stage of development at which IGF-1 has been shown to reduce negative effects of elevated temperature on embryos is at Day 5 after insemination [15,16]. Finally, it is possible that treatment with bST may not change the bioavailable IGF-1 in the follicular, oviductal or uterine environment sufficiently to protect oocytes or embryos from elevated temperature. Treatment of lactating cows with bST increased amounts of IGF-1 in follicular fluid but not in uterine flushings [28].

In Experiment 2, the proportion of cows that ovulated following the ovulation synchronization protocol tended to be greater for cows treated with bST. This possible effect of bST did not reflect a difference in luteolysis following administration of PGF_2α _because there was no difference between control and bST-treated cows in the proportion of cows with low progesterone concentrations on the putative day of ovulation (Day 0). Perhaps bST increased luteinizing hormone (LH) release in response to GnRH or increased the rate of follicular development so that preovulatory follicles were more likely to respond to LH. The literature is equivocal as to whether bST affects LH secretion [29,30] but it is well established that bST can enhance follicular growth [18].

## Conclusion

Results failed to indicate a beneficial effect of bST treatment on fertility of lactating dairy cows.

## Methods

### Effect of bST at insemination in cows in a temperate climate (Experiment 1)

The study was carried out at an 1100-cow dairy farm located in the central plateau of México (Municipio de El Marqués, Estado de Querétaro, 20.31° N 100.09° W). The climate is temperate and sub-humid, with rains during the summer and an average yearly temperature of 18.7°C. The study was conducted from July – December 2006. The average daily minimum and maximum dry bulb temperatures and average daily relative humidities during the study obtained from the Servicio Meteorológico Nacional [31] for El Marqués are shown in Table 4. Cows were milked twice daily and the average milk production per cow for the herd was 9800 kg per lactation. Cows were kept in a free-stall barn and fed a total mixed ration six times daily based on alfalfa hay, corn silage and concentrate according to National Research Council requirements [32].

**Table 4:** Average daily minimum and maximum dry bulb temperatures and average daily relative humidity during the experiments.

	Average daily minimum dry bulb temperature (°C)	Average maximum dry bulb temperature (°C)	Average daily relative humidity
Experiment 1 (Mexico)			
			
July	14.8	27.9	73%
August	15.4	27.0	70%
September	15.2	26.7	75%
October	13.4	26.3	73%
November	9.2	23.4	66%
December	7.2	22.7	61%

Experiment 2 (Florida)			
			
June	12.6	34.4	74%
July	13.4	35.4	75%
August	18.2	34.5	76%
September	10.9	33.7	76%
October	-0.7	31.3	70%
November	-0.4	29.6	74%

The protocol used procedures routinely performed on the dairy and approval from the local animal care committee was not required. All cows eligible for first service were considered for the study. Those diagnosed by a veterinarian with retained placenta, pyometra, cystic ovaries or endometritis or those with a BCS < 2.5 at artificial insemination using a scale of 1 to 5 [33] were excluded. Cows were paired according to insemination date and randomly assigned within pair to receive either a single injection of a sustained-release formulation of bST (n = 100; 500 mg, s.c.; Boostin® -S, Schering-Plough, México; also contains 1.8 g vitamin E) or a control injection (n = 100; 2 ml of a saline placebo) at first service. Injections were given by the investigators at the same time as when insemination using frozen-thawed semen was performed by a trained farm technician.

Estrus was observed from Day 50 postpartum onwards by direct observation continuously for 4-h periods in the morning and the evening. At ~Day 60 postpartum (range 56 to 64 days), cows were palpated per rectum. Cows with a corpus luteum were allowed to return to estrus naturally (n = 130) and were inseminated 12 h after detection of estrus. Cows without a corpus luteum or follicle > 10 mm in diameter were considered anestrus and excluded from the study. Cows without a corpus luteum and with a follicle >10 mm in diameter were subjected to the Ovsynch protocol (n = 70) [34]. Cows received gonadotropin releasing hormone (GnRH, 100 μg, i.m.; Ovalyse®, Pfizer, México, D.F., México) in the morning. Seven days later, in the morning, cows received a luteolytic dose of prostaglandin F_2α _(PGF_2α_, 500 μg i.m.; Celosil®, Schering-Plough, Xaltocan, Xochimilco, México) in the morning. Two days later, cows received a second injection of GnRH in the afternoon and cows were inseminated 16 h later. The average days in milk at insemination was 69 ± 14 days. Pregnancy diagnosis for cows not returning to estrus was performed by rectal palpation at ~45 days after insemination.

The proportion of inseminated cows that became pregnant at first service was analyzed by logistic regression with JMP6 software (JMP, Release 6. 2005, SAS Institute Inc., Cary, NC, USA). The analysis included effect of treatment, type of estrus (spontaneous or synchronized), parity (2 vs > 2) and days in milk (continuous).

### Effects of bST at insemination for cows in hot conditions (Experiment 2)

The experiment was conducted from June – November 2006 at a commercial 3000-cow dairy in Trenton, Florida, USA (29.36° N 82.49° W) utilizing 256 first-service, lactating Holstein cows. The climate is semi-tropical and humid. Meteorological measurements for the period of the study obtained from the Florida Automated Weather Network [35] for nearby Alachua, Florida are shown in Table 4. Cows were housed in a free-stall barn equipped with cooling fans and an automatic sprinkler system. Cows were milked two times daily in the morning and afternoon and fed as a group after each milking. The diet was a total mixed ration containing corn silage and alfalfa as the main ingredients with 77 Mcal of net energy for lactation/kg and 17.4% crude protein on a dry matter basis.

The experimental protocol was approved by the University of Florida Institutional Animal Care and Use Committee Cows (project E730). Cows were selected for the study because they were eligible for first service during the study period. Exclusions in Experiment 1 were not considered. A presynchronization-OvSynch protocol was utilized for first service TAI [36]. The protocol was initiated at an average of 37 days postpartum (range 30 to 50). For presynchronization, cows were administered 2 injections of 25 mg PGF_2α _(Pfizer Animal Health, New York, NY, USA, i.m.) at a 14-day interval. After another 14 days, an ovulation synchronization protocol was initiated consisting of 100 μg GnRH (Cystorelin, Merial Co., Athens, GA, USA, i.m.) followed 7 days later with 25 mg PGF_2α _i.m., and a second injection of GnRH (100 μg) at 72 h following PGF_2α _and coincident with TAI (at an average of 75 days postpartum). All injections were given in the morning. Several cows were observed in estrus and inseminated prior to completion of the synchronization protocol. These cows were excluded from the analysis. Cows observed in standing estrus post-TAI were re-inseminated and considered non-pregnant to the TAI. Pregnancy diagnosis for cows not returning into estrus was conducted by rectal palpation at an average of 45 days after TAI.

Cows were enrolled in the experiment each week (during a 12-week period during June – September) and were assigned randomly within each week to receive bST or serve as controls. Treatment with a sustained-release formulation of bST (500 mg, s.c.; Posilac®, Monsanto Co., St Louis, MO, USA) was performed at the same time as the last PGF_2α_injection of the synchronization protocol. Treatment was administered by the researchers in the depression on either side of the tail head. Control cows received no injection.

Body condition score was recorded for control and bST-treated cows at insemination using a scale of 1 to 5 in 0.25 increments [33]. Each week, a random subset of cows in each treatment was fitted with a temperature data logger (HOBO Water Temp Pro v1, Onset Computer Co., Bourne, MA, USA) attached to a blank control internal drug release insert (Pfizer Animal Health) that was inserted into the vagina. The data logger recorded vaginal temperature at 15-min intervals beginning at 1100 h on Day 4 post-TAI for 3 days. Data were downloaded from the data logger into a laptop computer after removal from the cow. Average vaginal temperature for each hour was calculated. A new subset of cows was enrolled each week so that vaginal temperatures were recorded from a total of 21 control cows and 21 bST-treated cows. Note that results of a previous experiment indicated that insertion of HOBO devices into the vagina for 7 days did not affect fertility [6].

Blood samples were collected from each cow at the time of insemination and at Day 7 after insemination to determine plasma progesterone concentration. Blood samples were collected by coccygeal venipuncture (artery or vein) into evacuated heparinized 10-mL tubes (Becton Dickinson, Franklin Lakes, NJ, USA). Following collection, blood samples were placed in an ice chest until further processing in the laboratory (within approximately 2 to 6 h). Blood samples were centrifuged for 15 min at 4°C at 3000 × g. Plasma was separated and stored at -20°C until assayed for progesterone concentration using a commercial radioimmunoassay (Coat-a-Count, Diagnostic Products Corporation, Los Angeles, CA, USA). The intra- and interassay coefficients of variance were 6% and 9%, respectively, and assay sensitivity was 0.11 ng/ml. Cows with low plasma progesterone concentrations (< 1 ng/ml) were considered to not have a functional corpus luteum while cows with high plasma progesterone concentrations (≥ 1 ng/ml) were considered to have a functional corpus luteum. Cows that had low progesterone concentration on Day 0 and high concentration on Day 7 were determined to be successfully synchronized in response to the ovulation synchronization regimen.

Data on the proportion of cows pregnant after TAI were analyzed with the LOGISTIC procedure of SAS using a backward stepwise logistic model. Data were analyzed for all cows and for the subset of cows that were successfully synchronized. Variables were continuously removed from the model by the Wald statistic criterion if the significance was greater than 0.20. The initial mathematical model included the effects of bST, sire, technician, month of insemination, presence of a HOBO device, BCS class (< 2.5, ≥ 2.5), bST × BCS class, and bST × month. The final model included main effects of bST and month of insemination. The AOR estimates and the 95% Wald confidence intervals from logistic regression were obtained from this analysis. The Wald chi-square statistic was used to determine the probability value for each main effect that remained in the reduced model (significance was considered as *p *< 0.05).

Effects on the proportion of cows that were successfully synchronized for TAI was also analyzed with the LOGISTIC procedure of SAS using a backward stepwise logistic model as described above. The final model included main effects of bST and month of insemination.

Treatment effects on BCS and vaginal temperature were analyzed using least-squares analysis of variance with the GLM procedure of SAS. Tests of significance were made using the appropriate error terms based on calculation of expected means squares. Cow was a random effect and other main effects were considered fixed. The model for effects on BCS included month of insemination, bST and the interaction. The model for effects on vaginal temperature included bST, cow (bST), day, time of day, and all two way interactions.

## Abbreviations

AOR: adjusted odds ratio; BCS: body condition score; bST: bovine somatotropin; CI: 95% confidence interval; GnRH: gonadotropin releasing hormone; IGF-1: insulin-like growth factor-1; LH: luteinizing hormone; NS: non-significant; PGF_2α_: prostaglandin F_2α_; TAI: timed artificial insemination.

## Competing interests

The authors received no financial support or donation of materials from any commercial company. Moreover, the authors have no competing interests.

## Authors' contributions

All authors contributed to the experimental design and interpretation of experiments. AB, OAR, LAdeC, MBP and PJH administered treatments and collected data and JH-C, CCG, PJH and ADV oversaw the projects.

## References

[B1] Soderholm CG, Otterby DE, Linn JG, Ehle FR, Wheaton JE, Hansen WP, Annexstad RJ (1988). Effects of recombinant bovine somatotropin on milk production, body composition, and physiological parameters. J Dairy Sci.

[B2] Zoa-Mboe A, Head HH, Bachman KC, Baccari F, Wilcox CJ (1989). Effects of bovine somatotropin on milk yield and composition, dry matter intake, and some physiological functions of Holstein dairy cows during heat stress. J Dairy Sci.

[B3] West JW, Mullinix BG, Johnson JC, Ash KA, Taylor VN (1990). Effects of bovine somatotropin on dry matter intake, milk yield, and body temperature in Holstein and Jersey cows during heat stress. J Dairy Sci.

[B4] Jenny BF, Grimes LW, Pardue FE, Rock DW, Patterson DL (1992). Lactational response of Jersey cows to bovine somatotropin administered daily or in a sustained-release formulation. J Dairy Sci.

[B5] Elvinger F, Natzke RP, Hansen PJ (1992). Interactions of heat stress and bovine somatotropin affecting physiology and immunology of lactating cows. J Dairy Sci.

[B6] Jousan FD, de Castro e Paula LA, Block J, Hansen PJ (2007). Fertility of lactating dairy cows administered recombinant bovine somatotropin during heat stress. J Dairy Sci.

[B7] Esteban E, Kass PH, Weaver LD, Rowe JD, Holmberg CA, Franti CE, Troutt HF (1994). Interval from calving to conception in high producting dairy cows treated with recombinant bovine somatotropin. J Dairy Sci.

[B8] Esteban E, Kass PH, Weaver LD, Rowe JD, Holmberg CA, Franti CE, Troutt HF (1994). Reproductive performance in high producing dairy cows treated with recombinant bovine somatotropin. J Dairy Sci.

[B9] Moreira F, Risco CA, Pires MFA, Ambrose JD, Drost M, Thatcher WW (2000). Use of bovine somatotropin in lactating dairy cows receiving timed artificial insemination. J Dairy Sci.

[B10] Moreira F, Orlandi C, Risco CA, Mattos R, Lopes F, Thatcher WW (2001). Effects of presynchronization and bovine somatotropin on pregnancy rates to a timed artificial insemination protocol in lactating dairy cows. J Dairy Sci.

[B11] Santos JEP, Juchem SO, Cerri RLA, Galvao KN, Chebel RC, Thatcher WW, Dei CS, Bilby CR (2004). Effect of bST and reproductive management on reproductive performance of Holstein dairy cows. J Dairy Sci.

[B12] Blevins CA, Shirley JE, Stevenson JS (2006). Milking frequency, estradiol cypionate, and somatotropin influence lactation and reproduction in dairy cows. J Dairy Sci.

[B13] Morales-Roura JS, Zarco L, Hernández-Céron J, Rodríguez G (2001). Effect of short term treatment with bovine somatotropin at estrus on conception rate and luteal function of repeat breeding dairy cows. Theriogenology.

[B14] McGuire MA, Vicini JL, Bauman DE, Veenhuizen JJ (1992). Insulin-like growth factors and binding proteins in ruminants and their nutritional regulation. J Anim Sci.

[B15] Jousan FD, Hansen PJ (2004). Insulin-like growth factor-I as a survival factor for the bovine preimplantation embryo exposed to heat shock. Biol Reprod.

[B16] Jousan FD, Hansen PJ (2007). Insulin-like growth factor-I promotes resistance of bovine preimplantation embryos to heat shock through actions independent of its anti-apoptotic actions requiring PI3K signaling. Mol Reprod Dev.

[B17] Block J, Drost M, Monson RL, Rutledge JJ, Rivera RM, Paula-Lopes FF, Ocon OM, Krininger CE, Liu J, Hansen PJ (2003). Use of insulin-like growth factor-I during embryo culture and treatment of recipients with gonadotropin-releasing hormone to increase pregnancy rates following the transfer of in vitro-produced embryos to heat-stressed, lactating dairy cows. J Anim Sci.

[B18] Block J, Hansen PJ (2007). Interaction between season and culture with insulin-like growth factor-I on survival of in-vitro produced embryos following transfer to lactating dairy cows. Theriogenology.

[B19] Lucy MC (2000). Regulation of ovarian follicular growth by somatotropin and insulin-like growth factors in cattle. J Dairy Sci.

[B20] Quirk SM, Cowan RG, Harman RM, Hu CL, Porter DA (2004). Ovarian follicular growth and atresia: the relationship between cell proliferation and survival. J Anim Sci.

[B21] Nuttinck F, Charpigny G, Mermillod P, Loosfelt H, Meduri G, Freret S, Grimard B, Heyman Y (2004). Expression of components of the insulin-like growth factor system and gonadotropin receptors in bovine cumulus-oocyte complexes during oocyte maturation. Domest Anim Endocrinol.

[B22] Wasielak M, Bogacki M (2007). Apoptosis inhibition by insulin-like growth factor (IGF)-I during in vitro maturation of bovine oocytes. J Reprod Dev.

[B23] Moreira F, Paula-Lopes FF, Hansen PJ, Badinga L, Thatcher WW (2002). Effects of growth hormone and insulin-like growth factor-I on development of in vitro derived bovine embryos. Theriogenology.

[B24] Kölle S, Stojkovic M, Reese S, Reichenbach HD, Wolf E, Sinowatz F (2004). Effects of growth hormone on the ultrastructure of bovine preimplantation embryos. Cell Tissue Res.

[B25] Ealy AD, Aréchiga CF, Bray DR, Risco CA, Hansen PJ (1994). Effectiveness of short-term cooling and vitamin E for alleviation of infertility induced by heat stress in dairy cows. J Dairy Sci.

[B26] Bilby CR, Bader JF, Salfen BE, Youngquist RS, Murphy CN, Garverick HA, Crooker BA, Lucy MC (1999). Plasma GH, IGF-I, and conception rate in cattle treated with low doses of recombinant bovine GH. Theriogenology.

[B27] Hansen PJ (2007). Exploitation of genetic and physiological determinants of embryonic resistance to elevated temperature to improve embryonic survival in dairy cattle during heat stress. Theriogenology.

[B28] Lucy MC, Thatcher WW, Collier RJ, Simmen FA, Ko Y, Savio JD, Badinga L (1995). Effects of somatotropin on the conceptus, uterus, and ovary during maternal recognition of pregnancy in cattle. Dom Anim Endocrinol.

[B29] Dalton JC, Marcinkowski DP (1994). Effect of sometribove administration on LH concentration in dairy cattle. Theriogenology.

[B30] Schemm SR, Deaver DR, Griel LC, Mueller LD (1990). Effects of recombinant bovine somatotropin on luteinizing hormone and ovarian function in lactating dairy cows. Biol Reprod.

[B31] Servicio Meteorológico Nacional. http://smn.cna.gob.mx/.

[B32] National Research Council (2001). Nutrient Requirements of Dairy Cattle.

[B33] Edmonson AJ, Lean IJ, Weaver LD, Farver T, Webster G (1989). A body condition scoring chart for Holstein dairy cows. J Dairy Sci.

[B34] Pursley JR, Wiltbank MC, Stevenson JS, Ottobre JS, Garverick HA, Anderson LL (1997). Pregnancy rates per artificial insemination for cows and heifers inseminated at a synchronized ovulation or synchronized estrus. J Dairy Sci.

[B35] Florida Automated Weather Network. http://fawn.ifas.ufl.edu.

[B36] Portaluppi MA, Stevenson JS (2005). Pregnancy rates in lactating dairy cows after presynchronization of estrous cycles and variations of the Ovsynch protocol. J Dairy Sci.

